# A case report on the role of endoscopic retrograde cholangiopancreatography in the diagnosis of hemosuccus pancreaticus

**DOI:** 10.1097/MD.0000000000031561

**Published:** 2022-11-11

**Authors:** Sung Mi Moon, Kyu-Hyun Paik, Ji Chang Kim, Won Suk Park

**Affiliations:** a Division of Gastroenterology, Department of Internal Medicine, Daejeon St. Mary’s Hospital, College of Medicine, The Catholic University of Korea, Jung-gu, Daejeon, Republic of Korea; b Department of Radiology, Daejeon St. Mary’s Hospital, College of Medicine, The Catholic University of Korea, Jung-gu, Daejeon, Republic of Korea.

**Keywords:** case report, ERCP, gastrointestinal hemorrhage, hemosuccus pancreaticus, multidisciplinary team

## Abstract

Hemosuccus pancreaticus (HP) is a rare cause of gastrointestinal bleeding that sometimes leads to life-threatening conditions. However, the diagnosis of HP is often delayed due to its rarity and intermittent signs of bleeding, making it challenging to determine the source of bleeding in some patients.

**Patient concerns:**

An 83-years-old man was transferred to our hospital for evaluation of the source of intermittent upper gastrointestinal bleeding involving melena and worsening anemia.

**Diagnosis:**

HP was diagnosed via endoscopic retrograde cholangiopancreatography (ERCP) and sequential angiography using a multidisciplinary approach.

**Interventions:**

Initial upper and lower gastrointestinal endoscopies did not reveal any source of bleeding. Emergency upper endoscopy performed when the patient had hematochezia and hypotension confirmed a spurt of bleeding from the major duodenal papilla. However, contrast-enhanced computed tomography and angiography could not identify the source of the bleeding from the major duodenal papilla. ERCP for inducing bleeding from the source and indicating the bleeding point was performed according to the decision of the multidisciplinary team. Immediately thereafter, sequential angiography was performed and HP, due to the rupture of a pseudoaneurysm of the splenic artery, was diagnosed. As a result, surgical resection of the pancreas could be avoided by accurately embolizing the bleeding focus of HP using a multidisciplinary team approach.

**Outcomes:**

The patient was discharged in a hemodynamically stable condition. There was no further gastrointestinal bleeding or procedure-related complication until 6 months after discharge.

**Lessons:**

HP should be considered by endoscopists during the differential diagnosis of intermittent upper gastrointestinal bleeding in patients with a history of pancreatitis. A multidisciplinary team approach is an effective method to determine the source or location of bleeding, which may reduce mortality and morbidity by avoiding additional pancreatectomies.

## 1. Introduction

Hemosuccus pancreaticus (HP) is defined as a condition in which blood originating from the pancreas or adjacent structures flows out of the major duodenal papilla via the pancreatic duct. It is very difficult to diagnose HP as it is a rare cause of upper gastrointestinal bleeding, and the nature of bleeding is intermittent.^[1]^ Moreover, even if HP is revealed by upper endoscopy, the difficulty in determining the location of the bleeding source results in treatment delay, which can become a life-threatening condition.^[2]^ Contrast-enhanced computed tomography (CT) helps demonstrate pancreatic disease and complications in adjacent structures, such as pseudoaneurysms or pseudocysts. Angiography is the gold standard for the diagnosis of pseudoaneurysms, with a sensitivity of up to 96 %,^[3]^ and for its treatment, particularly in hemodynamically stable patients or in those unfit for surgery. However, if CT and angiography show negative results for the bleeding source, treatment will inevitably be delayed, and the patient may be in a life-threatening situation.

To the best of our knowledge, there have been no reports of cases in which HP was diagnosed via endoscopic retrograde cholangiopancreatography (ERCP) and sequential angiography using a multidisciplinary approach. Herein, we report a case of HP caused by a pseudoaneurysm rupture of the splenic artery that presented with intermittent gastrointestinal bleeding. The location of the bleeding source was identified using sequential angiography after ERCP. ERCP plays a key role in inducing bleeding by insertion of a guidewire into the pancreatic duct.

## 2. Case presentation

An 83-years-old man was transferred to our hospital for evaluation of intermittent upper gastrointestinal bleeding involving melena and worsening anemia. Three years ago, the patient underwent ERCP for the treatment of acute pancreatitis caused by a pancreatic duct stone. The patient first presented with melena 2 months ago; this was followed by intermittent melena accompanied by worsening anemia. Initial upper endoscopy showed a few old blood clots in the stomach and duodenum, but the source of the upper gastrointestinal bleeding could not be identified. He was taking 100 mg aspirin and medicines for diabetes and hypertension daily. Although the patient abused alcohol, he did not smoke and had no significant family medical history.

**Figure 1.**
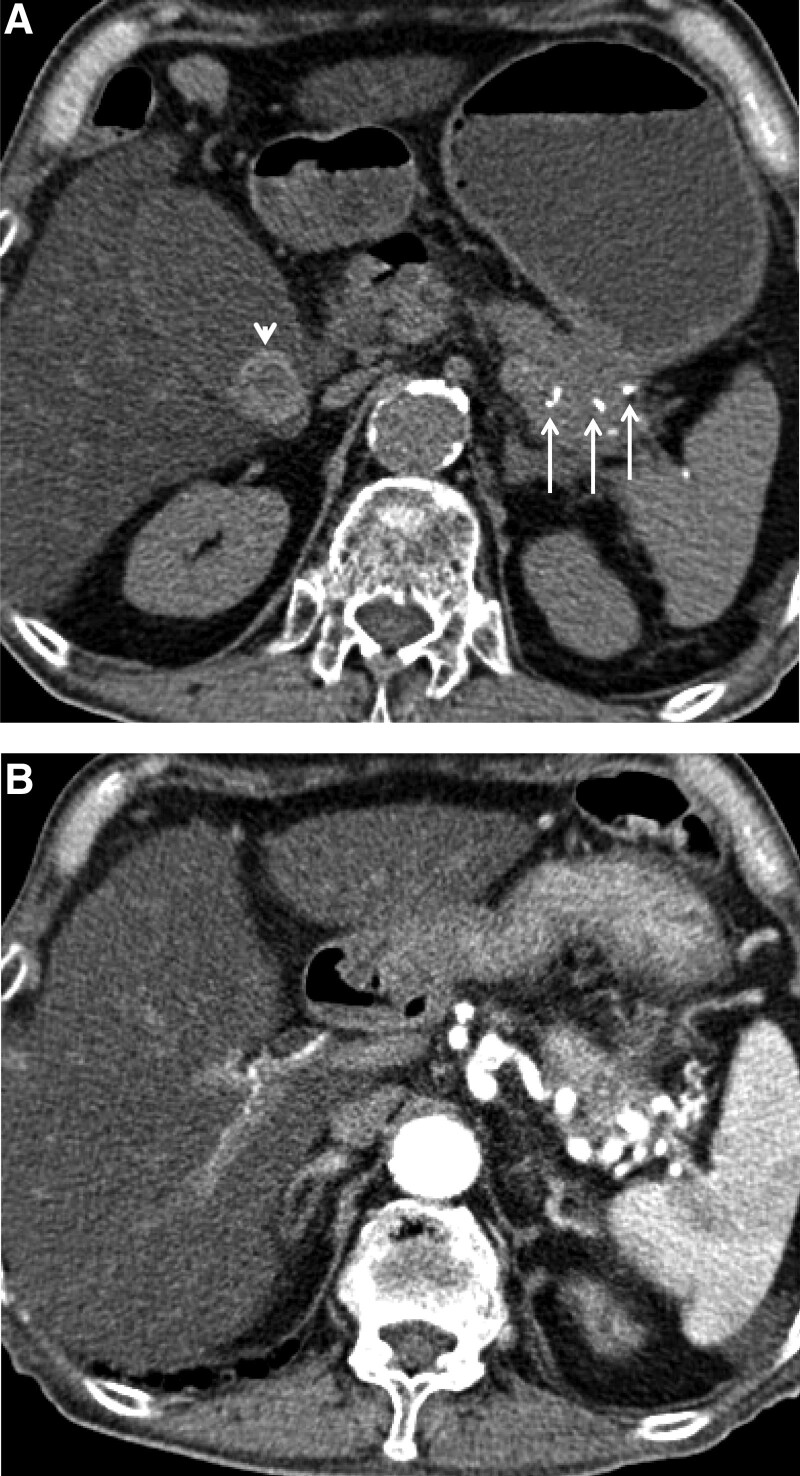
Contrast-enhanced computed tomography (CT) scans. A: Pre-contrast phase CT scan showing a gallbladder stone (arrowhead) and pancreatic calcifications (arrow); B: Arterial phase CT scan showing the tortuosity of the splenic artery. CT = computed tomography.

On admission, the patient’s vital signs were stable, with a blood pressure of 130/80 mm Hg, pulse rate of 82 beats/minute, and body temperature of 36.5 °C. Physical examination revealed a poor performance status and conjunctival pallor. The abdomen was soft and undistended. Other physical examinations were unremarkable. The results of a complete blood count test revealed a hemoglobin of 6.4 g/dL, hematocrit of 19%, mean corpuscular volume of 108.2 fL, mean corpuscular hemoglobin of 36.3 pg, and mean corpuscular hemoglobin concentration of 33.6%. The results of the blood chemistry tests were within normal reference limits: total bilirubin of 0.6 mg/dL (reference range 0.2–1.2 mg/dL), aspartate aminotransferase of 27 IU/L (reference range 8–40 IU/L), alanine aminotransferase of 9 IU/L (reference range 5–41 IU/L), alkaline phosphatase of 100 IU/L (reference rage 40–130 IU/L), serum amylase of 77 IU/L (reference range 41–134 IU/L), and serum lipase of 39 IU/L (reference range 13–60 IU/L). In the emergency room, initial upper and lower gastrointestinal endoscopies did not reveal the source of bleeding. The pre-contrast phase CT scans showed severe fatty changes in the liver, gallbladder stones, and multiple calcifications at the pancreatic tail (Fig. 1A). Arterial phase CT scans showed peripancreatic inflammation at the tail of the pancreas and a tortuous splenic artery (Fig. 1B), without a definite source of bleeding.

**Figure 2.**
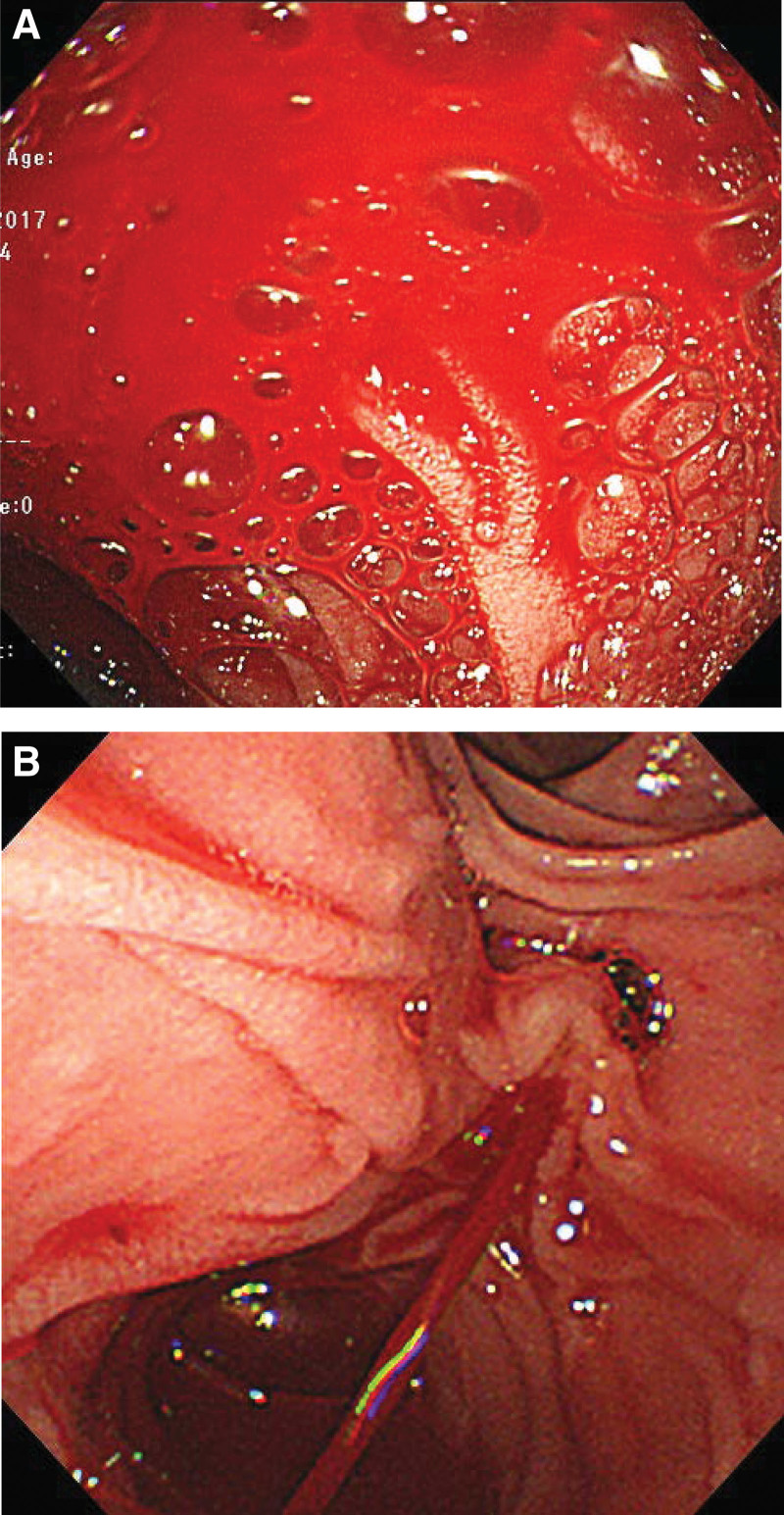
Endoscopic finding. A: Emergency endoscopy showing a large amount of fresh blood in the duodenum; B: Duodenoscopy showing blood spurting from the major duodenal papilla.

**Figure 3.**
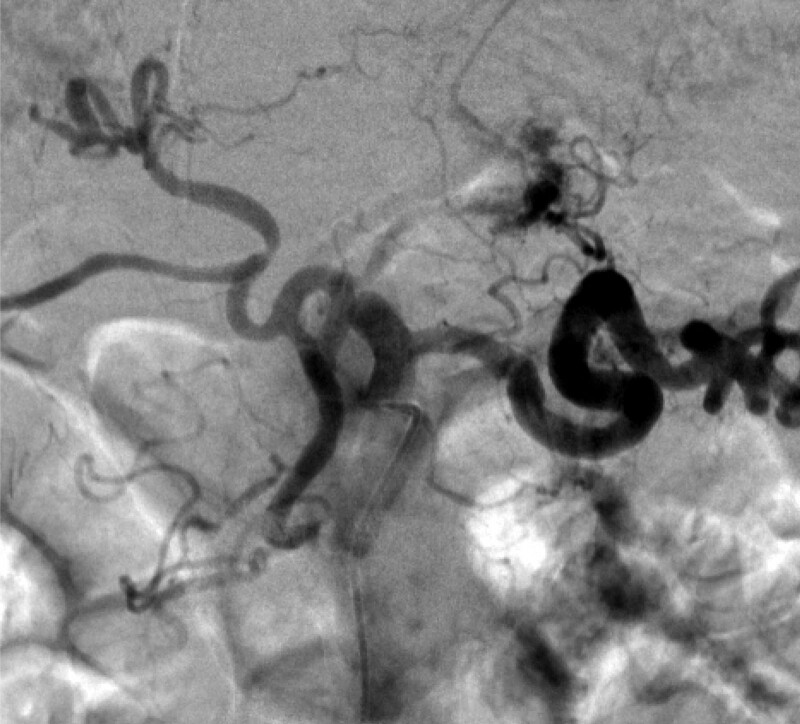
Angiographic finding. A celiac artery angiogram showing a tortuous splenic artery without a definite source of bleeding.

**Figure 4.**
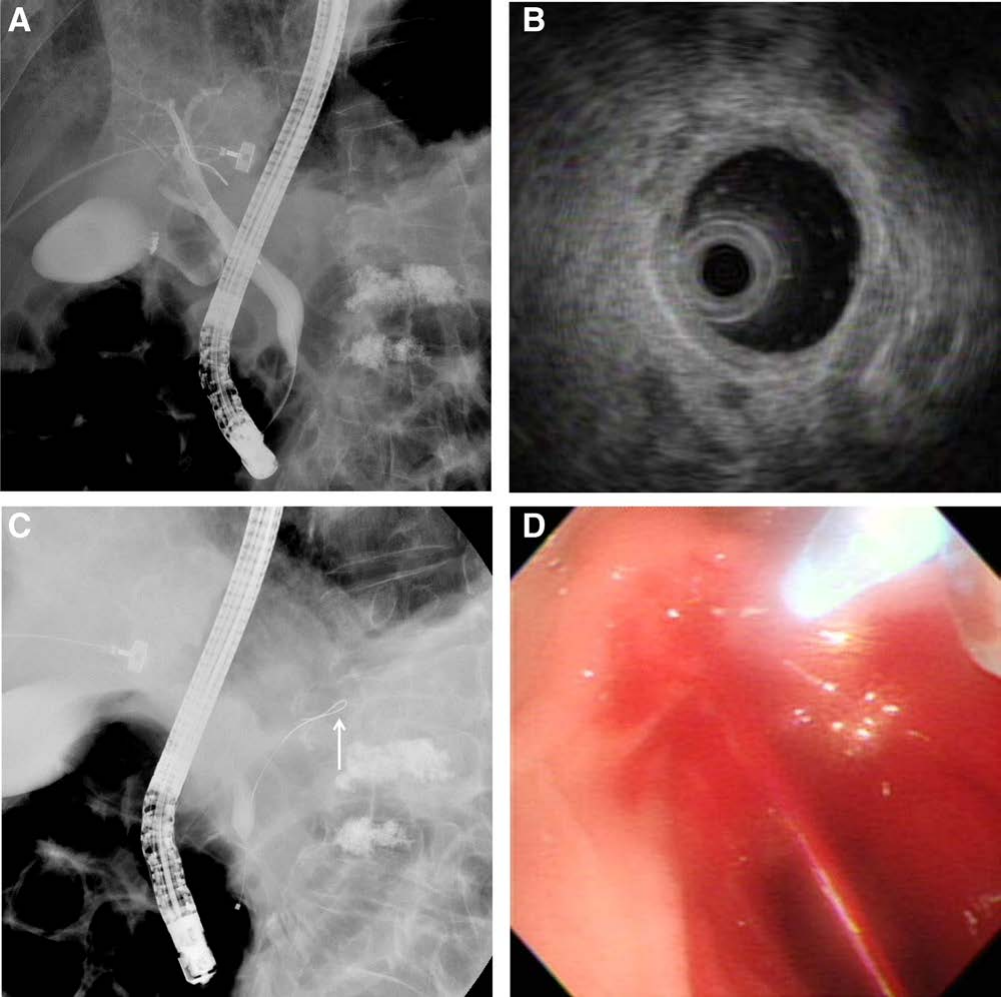
Endoscopic retrograde cholangiopancreatography (ERCP) and intraductal ultrasonography (IDUS) findings. A: ERCP image showing the common bile duct without abnormal filling defects. B: IDUS cross-sectional imaging showing the normal layers of the common bile duct wall; C: The position of the bent tip of a guidewire (arrow) inserted into the main pancreatic duct at the onset of a blood spurt from the major duodenal papilla is observed (IDUS); D: Duodenoscopy showing blood spurting from the major duodenal papilla following a pancreatic duct guidewire placement (IDUS). ERCP = endoscopic retrograde cholangiopancreatography, IDUS = Intraductal ultrasonography.

**Figure 5.**
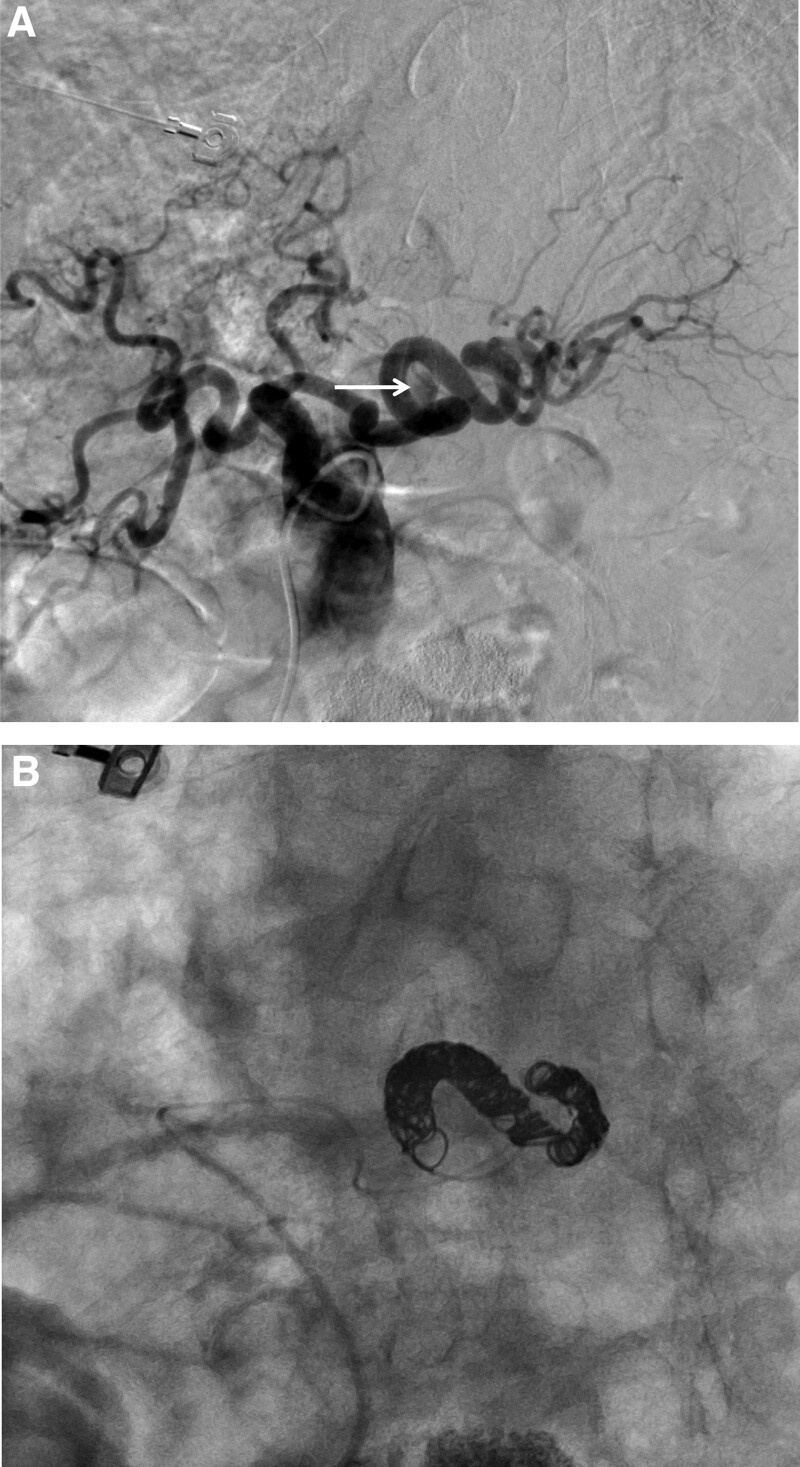
Angiography findings by a multidisciplinary team approach. A: A celiac artery angiogram showing a small pseudoaneurysm at the mid portion of the tortuous splenic artery (arrow); B: Microcoil embolization of the splenic artery pseudoaneurysm was performed.

The patient received a transfusion of red blood cells, and his treatment was planned conservatively. On day 4 after admission, the patient suddenly presented with hematochezia and hypotension. Emergency endoscopy revealed a large amount of fresh blood in the duodenum (Fig. 2A) and a blood spurt coming from the major duodenal papilla (Fig. 2B). However, a subsequent emergency angiography (Fig. 3) failed to localize the causative vessel of the blood spurt from the major duodenal papilla. Fortunately, the patient’s vital signs stabilized with transfusion and conservative management. A multidisciplinary team first planned for an endoscopist to perform ERCP to determine the exact location from where the blood spurt was triggered and then for an interventional radiologist to perform angiography immediately following bleeding. To rule out the possibility of bleeding from the bile duct, endoscopic retrograde cholangiography (Fig. 4A) and intraductal ultrasonography (IDUS) (Fig. 4B) were performed. This revealed no evidence of bleeding in the bile duct. Next, pancreatic duct guidewire placement was performed to induce bleeding in the pancreatic duct. In a detailed manner, the tip of the guidewire was curved into a U-shape (Fig. 4C) and pushed into the pancreatic duct slowly to the point where the bleeding from the major duodenal papilla occurred, and this was observed using fluoroscopic imaging. In this manner, we were able to successfully induce a blood spurt from the major duodenal papilla (Fig. 4D) and specify the exact point of bleeding using fluoroscopy (Fig. 4). Angiography was immediately performed by an interventional radiologist, which revealed a tortuous splenic artery as well as a definite small pseudoaneurysm around the location of the blood spurt during ERCP (Fig. 5A). Transarterial catheter embolization using microcoils was performed successfully for the pseudoaneurysm originating from the splenic artery (Fig. 5B).

The patient was discharged in a hemodynamically stable condition. After 4 weeks, follow-up results of a complete blood count test were a hemoglobin of 13.4 g/dL and hematocrit of 41.7%. There was no further gastrointestinal bleeding or procedure-related complication until 6 months after discharge.

## 3. Discussion

HP refers to gastrointestinal bleeding in the major duodenal papilla through the pancreatic duct. This condition is one of the rarest causes of upper gastrointestinal bleeding and is mostly associated with acute or chronic pancreatitis.^[1,4]^ The most common source of bleeding in HP is pseudoaneurysm rupture, which is a complication of acute or chronic pancreatitis. However, it is sometimes reported that the bleeding from pancreatic pseudocysts; pancreatic tumors, such as pancreatic carcinoma, serous cystic neoplasm, mucinous cystic neoplasm, and neuroendocrine tumor; pancreas divisum; and pancreatic trauma may lead to HP.^[1,5]^ The involved artery is mostly the splenic artery, although the common hepatic, gastroduodenal, and pancreaticoduodenal arteries may also rarely be involved.^[2]^ In this case, the patient had acute pancreatitis 3 years prior and underwent ERCP and pancreatic duct stenting for the treatment of a pancreatic duct stone. The source of bleeding was a pseudoaneurysm rupture of the tortuous splenic artery along the course of the pancreas. In this case, it was questionable whether the pseudoaneurysm was the result of erosion of the artery wall by pancreatic duct stenting or autodigestion of the artery by pancreatitis itself.

Patients with HP have a classic triad of abdominal pain, gastrointestinal bleeding including hematemesis and melena, and increased levels of pancreatic enzymes.^[6]^ Abdominal pain and gastrointestinal bleeding are intermittent in patients with HP. This intermittency is presumed to be due to alternating formation and dissolution of blood clots in the pancreatic duct.^[6–8]^ Therefore, abdominal pain has a repetitive crescendo-decrescendo nature in approximately half of patients with HP^[8]^; this is caused by increased intraductal pressure due to blood clots in the pancreatic duct.^[9]^ Intermittent melena, which is accompanied by worsening anemia, is the most common symptom of HP, although there are some cases of hematemesis in patients with HP.^[6,10,11]^ Owing to the intermittent nature of bleeding, HP cannot be diagnosed by upper endoscopic evaluations in approximately half of patients.^[10,12]^ In our case, the patient had only intermittent melena accompanied by worsening anemia, without abdominal pain or increased serum amylase levels. In addition, several sessions of upper endoscopy could not identify the source of the bleeding, which made it difficult for HP to be diagnosed initially.

Contrast-enhanced CT is an excellent modality for demonstrating the features of pseudoaneurysms, such as the sentinel clot on pre-contrast CT scans and simultaneous opacification of an aneurysmal artery on arterial phase CT scans.^[4]^ Angiography is the gold standard for identifying the causative artery and providing therapeutic intervention.^[4]^ However, in a report by Rammohan et al, 10% of cases of HP had negative results on CT and 11% had negative results on angiography.^[10]^ In this case, contrast-enhanced CT showed chronic pancreatitis and a tortuous splenic artery, suggesting that HP was caused by a splenic artery pseudoaneurysm. However, we could not demonstrate the pseudoaneurysm rupture on contrast-enhanced CT or confirm the splenic artery pseudoaneurysm on angiography. In this case, there were also concerns regarding the possibility that the source of bleeding was not the pancreatic duct but the bile duct. Therefore, our multidisciplinary team planned to perform IDUS along with endoscopic retrograde cholangiography to differentiate hemobilia from HP and to perform intentional pancreatic duct guidewire placement to destroy preformed clots. This was followed by sequential angiography performed by an interventional radiologist to locate and treat the bleeding sources.

IDUS examination along with ERCP is a useful diagnostic tool for detecting abnormal lesions in both the bile and pancreatic ducts. As shown in this case, IDUS examination helps in ruling out hemobilia originating from the bile duct mucosa by providing precise cross-sectional images. Moreover, using ERCP, the plan to destroy pre-formed clots in the pancreatic duct by stepwise insertion of a guidewire under fluoroscopic imaging played a conclusive role in confirming the diagnosis of HP and determining the source of bleeding in the patient.

There are 2 therapeutic options for HP. First is endovascular treatment, including coil embolization and covered stent grafting.^[1,13,14]^ All hemodynamically stable patients with HP should undergo initial angiographic evaluation with embolization if possible. Second is pancreatic resection; this should be considered as an alternative if the interventional procedure fails or is unavailable or when rebleeding occurs. However, if a patient is not eligible for surgical treatment due to old age, comorbidities, or poor performance status, it is essential to identify and treat the exact location of the bleeding source using the angiographic intervention. The authors believe that a multidisciplinary team approach would be the most effective method to resolve the situation in which the source of bleeding is unclear in patients with HP.

## 4. Conclusions

HP should be considered by endoscopists during the differential diagnosis of intermittent upper gastrointestinal bleeding in patients with a history of pancreatitis. A multidisciplinary team approach is an effective method to determine the source or location of bleeding, which may reduce mortality and morbidity by avoiding additional pancreatectomies. Herein, this research reports a case of HP in which the bleeding source was identified and successfully treated using sequential angiography following ERCP.

## Acknowledgements

The authors thank Professor Drs In Seok Lee MD (Seoul St. Mary’s Hospital, The Catholic University of Korea), Seung Ok Lee, and Soo Teik Lee (Chonbuk National University Medical School) for their outstanding teaching and advice.

We would like to thank Editage (www.editage.co.kr) for English language editing.

## Author contributions

**Conceptualization:** Sung Mi Moon, Ji Chang Kim, Won Suk Park.

**Investigation:** Ji Chang Kim.

**Supervision:** Won Suk Park.

**Writing – original draft:** Sung Mi Moon, Won Suk Park.

**Writing – review & editing:** Kyu-Hyun Paik, Won Suk Park.
